# An efficient model of human endometriosis by induced unopposed estrogenicity in baboons

**DOI:** 10.18632/oncotarget.7516

**Published:** 2016-02-19

**Authors:** Hareesh B. Nair, Robert Baker, Michael A. Owston, Renee Escalona, Edward J. Dick, John L. VandeBerg, Klaus J. Nickisch

**Affiliations:** ^1^ Evestra, Inc., San Antonio, TX, USA; ^2^ Southwest National Primate Research Center, Texas Biomedical Research Institute, San Antonio, TX, USA

**Keywords:** endometriosis, baboon, endometrial stromal cells, attachment, invasion, unopposed estrogenicity, Pathology Section

## Abstract

Endometriosis is a chronic estrogen-dependent disease that occurs in approximately 10% of reproductive age women. Baboons offer a clear benefit for studying the initiation and progression of endometriosis since baboon is very close to humans phylogenetically. Progestins are used in the treatment of endometriosis. The therapeutic window of progestins depends on the ratio of its affinity towards progesterone receptor agonism verses antagonism. The present study is to determine the role of pure antiprogestin in baboon endometriosis. We hypothesize that pure antiprogestin will induce unopposed estrogenicity and spontaneous endometriosis in baboons. The rate of endometrial invasion and attachment through modeled peritoneum in the presence and absence of progesterone and antiprogestin was evaluated in this study. A baboon model of endometriosis induced by unopposed estrogenicity using progesterone receptor antagonist (EC304) was used in this study. We observed EC304 has induced unopposed estrogenicity that deregulated proteins involved in attachment, invasion, cell growth, and steroid hormone receptors in this model. Our data suggest that depleting progesterone levels in the endometrium will increase estrogen hyper-responsiveness that leads to increased endometriotic lesion progression in the baboon (*Papio anubis)* model. This study reports a refined model of human endometriosis in baboons that could potentially be used to develop new diagnostic and therapeutic strategies for the benefit of women suffering from endometriosis.

## INTRODUCTION

Endometriosis is a chronic estrogen-dependent disease that occurs in approximately 10% of reproductive age women [1, 2]. Endometriosis results from the presence of endometrial glands and stroma outside of the normal location in the uterus [3, 4]. The main clinical features associated with endometriosis are chronic pelvic pain, pain during intercourse and infertility [5, 6, 7]. Patients diagnosed with endometriosis have reduced rates of follicular growth, limited functional capacity of the pre-ovulatory follicle, aberrant fertilization rates, abnormal pre-implantation embryonic development, derailed early luteal function and reduced implantation rates [8, 9]. Endometriosis is often expensive to treat and very complex to study since there are notable delays in the diagnosis and variations in symptoms as well as disease progression in patients [10]. Also, at the time of clinical presentation, most women have established disease, making the initiation of endometriosis difficult to study.

No preventive drug therapy for endometriosis is available at the current time. All treatments are based on symptoms and target the induction of atrophy of the hormone dependent endometrium. The goal of therapy is to reduce the levels of estrogens to decrease the stimulatory effects on the endometrium while maintaining sufficient estrogen deficiency symptoms. The main treatment options include progestins, oral contraceptives and gonadotropin releasing hormone (GnRH) - antagonists. All of these treatment options seem to have comparable efficacy, but the rather unpredictable progress of the disease and side effects renders these therapies as suboptimal treatments [10, 11]. The main side effects of progestin therapies are interim bleeding, weight gain and reduced libido. Oral contraceptives are generally well tolerated but they are not as effective in reducing pain [12]. GnRH-agonists are highly effective but they lead to a significant reduction in bone density [13, 14].

Even though rodent models including nude mice have been developed for experimental endometriosis, the anatomy and physiology of the rodent endometrium do not apply to the human endometrium, and rodents do not develop endometriosis. The peritoneal environment in rodents is not identical that of humans, and lesions induced in rodents are not exactly comparable to endometriotic lesions in women [15, 16]. Rodent species display a short estrous cycle with an abbreviated luteal phase; they neither menstruate nor develop spontaneous endometriosis. Also they do not undergo decidualization without artificial manipulations that include exposure to exogenous progesterone [15, 17]. Currently, no available rodent models succeed in reproducing the effects of the disease on the human endometrium [18, 19]. Baboons offer a clear benefit for studying the initiation and progression of endometriosis, in part because baboons are very close to humans phylogenetically [19]. Moreover baboon reproductive anatomy and physiology are similar to those of humans, including menstrual cycle characteristics, embryo implantation, and fetal development [19, 20]. Major advantages of the baboon model in gynecologic research include the spontaneous presence of peritoneal fluid and the accessibility of the uterine cavity through the cervix, allowing endometrial sampling without hysterectomy. Spontaneous endometriosis in the baboon has been found to be similar to various stages of endometriosis in women [20, 21]. While the baboon is an excellent model to study the initiation and progression of endometriosis, prevalence of induced endometriosis using current methods is very limited and thus a major hindrance in using this model to test interventional drug candidates. In a recent study the incidence of induced endometriosis in baboons was only 27.6% (n=30) [16, 22].

The major drawbacks of current medical therapies of endometriosis are bleeding irregularities, vaginal dryness and also endometrial cancer [23]. Recently selective progesterone receptor modulators (SPRMs or mesoprogestins) have been proven to be very useful in the treatment of endometriosis [24]. SPRMs behave like progestins and progesterone antagonists (antiprogestins) show high-binding affinity to progesterone receptors. However in animal models SPRMs exhibited a different profile when compared to either progestins or antiprogestins. In the absence of progesterone, the SPRMs act like weak progestins and in the presence of progesterone, they may also show weak antiprogestagenic properties in some tissues, particularly in the endometrium [25].

In the present study, we have developed an efficient method to induce the endometriotic lesions in baboons using unopposed estrogenicity using antiprogestin treatment.

## RESULTS

### Effect of antiprogestin (EC304) in the attachment of endometrial cells on peritoneal mesothelium *in vitro*

**Figure 1:**
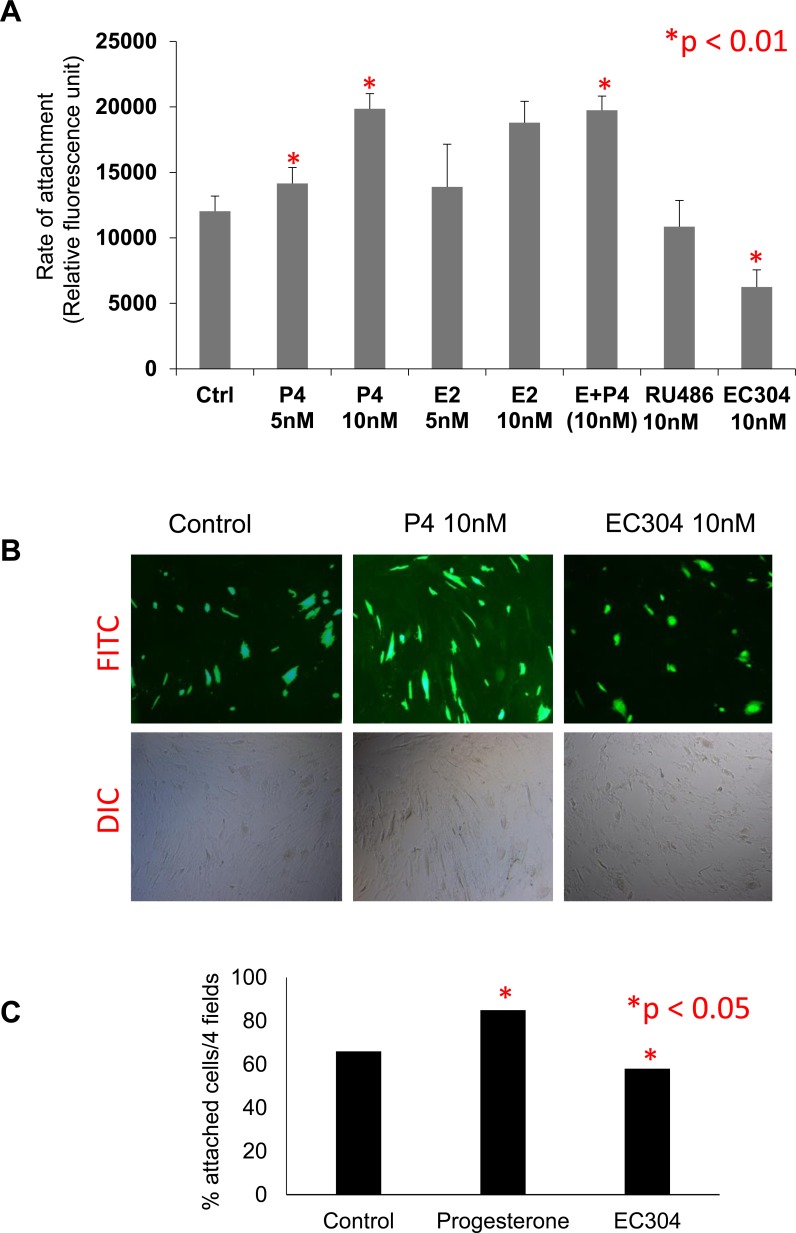
EC304 reduced endometrial epithelial cell attachment to peritoneal mesothelium *in vitro* **A.** Estrogen and progesterone dose dependently enhanced the attachment of HESC cells to monolayers of peritoneal mesothelial (LP9) cells (*p < 0.01). **B.** Fluorescently labeled HESCs attach on to LP cells at 6 h. EC304 shows reduced rate of attachment (*p < 0.05). **C.** The percent of attached HESCs were represented as compared with corresponding progesterone treated cells.

In order to determine the attachment of human endometrial stromal cells (HESEs) to peritoneal mesothelial cells (LP9), we determined the attachment of HESE on the monolayers of LP9 in the presence and absence of EC304 or progesterone. EC 304 was rationally designed and synthesized in our laboratory, and showed promising antiproliferative activity in breast cancer cells. EC304 was well characterized in receptor binding studies using select screen™ system and found strong antiprogestational effect with negligible antiglucocorticoid activity when compared to antiprogestin RU486 [28]. The attachment of HESCs in the presence of progesterone (5nM) was elevated when compared to control. The number of HESCs incrementally attached on to LP cells with 10nM progesterone. However, the cells treated with EC304 (10nM) as PR antagonist, show decrease in attachment when compared to control. The observed effect shows that progesterone increase the rate of attachment of menstrual endometrial stromal cells (HESCs) to peritoneal mesothelium (LP9) *in vitro* (Figure 1A, 1B, 1C). The decrease in the rate of attachment exerted by antiprogestin EC304 might be due the antiproliferative effect of this compound reported previously from our group [28].

### EC304 treatment did not change the rate of invasion of HESEs to peritoneal mesothelium

**Figure 2:**
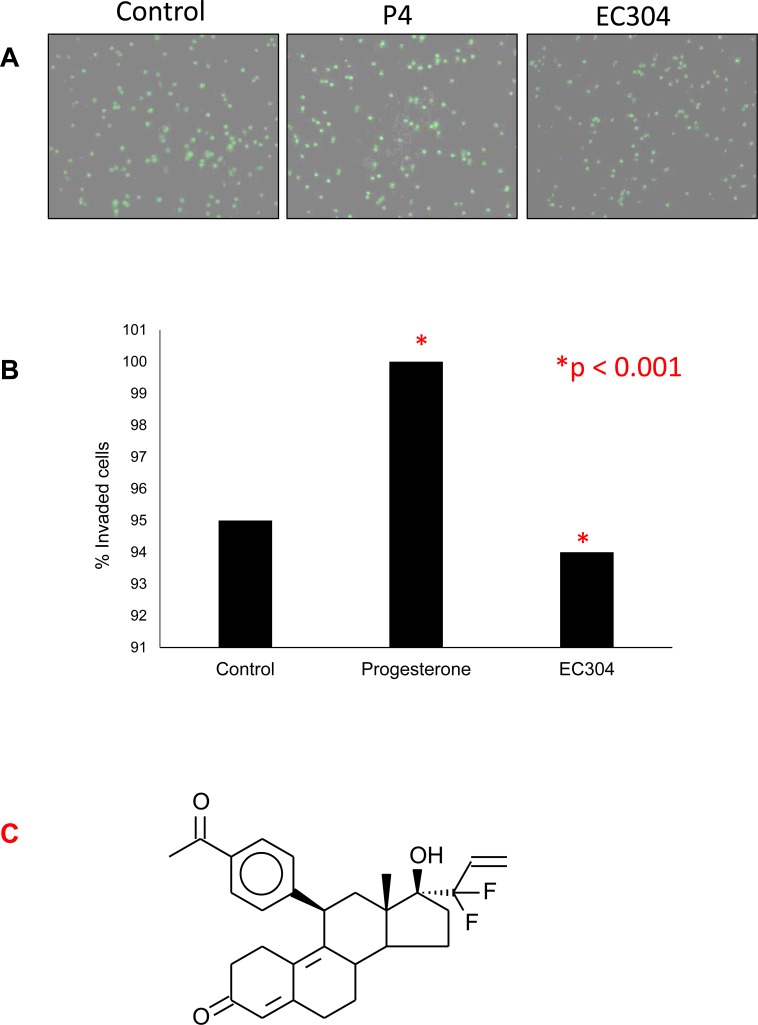
Trans-mesothelial invasion of HESCs over LP9 monolayers **A.** EC304 showed mild reduction in trans-mesothelial invasion of HESC cells. P4 induced invasion of HESCs though LP9 monolayers through the growth factor reduced matrigel. **B.** The percent of invaded cells through the LP9 monolayers were represented compared with control cells. **C.** Structure of EC304 (*P* < 0.001).

EC304 did not change the invasion of HESCs through monolayers of LP9 cells in the matrigel. The invasion of HESCs under progesterone (1nM) treatment increased the number of invaded cells when compared to control cells. With the treatment of EC304 (1, 10 nM) the cells seemed to be cytostatic and did not increase the rate of invasion through the matrigel, but rather showed a slight decrease in the invasion rate (Figure 2A, 2B). These results are in correlation with our previously published studies using EC304 in breast cancer cells [28]. The previously noted strong antiprogestational activity of EC304 might be responsible for the observed slight antiproliferative activity in reduced stromal cell invasion. Structure of EC304 is revealed in Figure 2C. The increased rate of invasion noted in progesterone alone treated cells is due to mitogenic potential of progesterone.

### Effect of antiprogestin (EC304) treatment on the development of baboon endometriosis

**Figure 3:**
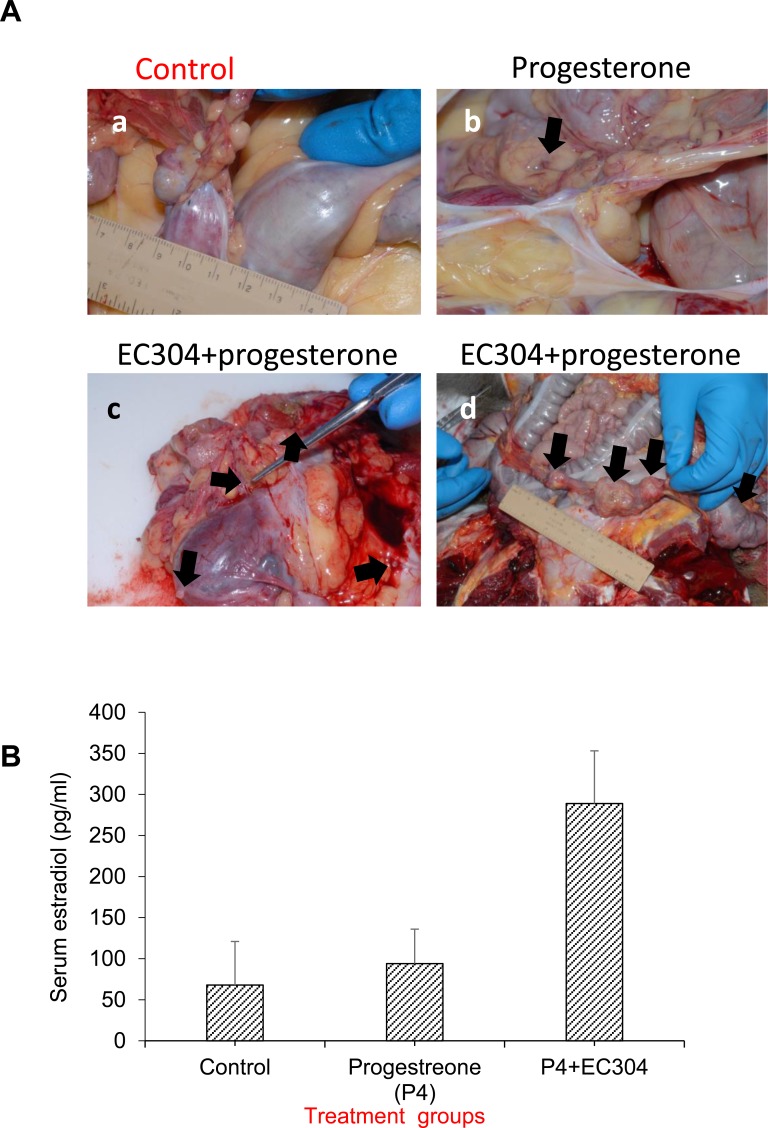
Antiprogestin (EC304) induced unopposed estrogenicity in baboons **A.** a- control and -b- Baboons treated with progesterone alone were essentially normal compared to control animals. c- First animal treated with progesterone+EC304: The serosa is infiltrated by glands (cysts with columnar epithelium and densely cellular stroma) and fibrous tissue; some of the glands and stroma infiltrate into the walls of the colon and uterus, and infiltrating as far as the submucosa of the colon. Endometriosis in serosa, oviduct, colon and uterus. d- Second animal treated with progesterone+EC304: There were multifocal accumulations of well differentiated endometrial glands, stroma and hemosiderin on the surface of the uterus, colon, and urinary bladder, as well as within the peritoneal cavity; large areas of hemorrhage and fibroplasia were also seen in these areas. **B.** EC304 treatment increased the serum estradiol (E2) levels in the baboons as result of induced unopposed etsrogenicity. (Using baboons as the model, this study make it expensive. Hence we regret to that preforming study in large groups of animals to get statistical significance is not feasible and consider as a pilot experiment).

Histologically confirmed endometriosis occurred in both animals treated with EC304 +progesterone, one of two animals treated with progesterone alone, and in the control animal. Lesions were generally more severe and extensive in the EC304 +progesterone treated animals. Grossly, there were variably extensive adhesions between the omentum, uterus, ovary, oviduct, colon, and urinary bladder. Occasional dark (gunpowder lesions) or orange-tan foci, generally less than 2mm diameter were seen in the adhesions and on serosal surfaces. Larger masses, up to 5 cm diameter were observed in the EC304 +progesterone treated animals. Histologically the lesions were characterized by accumulations of well differentiated endometrial glands, stroma and hemosiderin in the mesentery and on the serosal surfaces of the affected organs (Figure 3A).

### EC304 induced strong unopposed estrogenicity in baboon endometrium

The animals treated with potent antiprogestin EC304 (n=2) expressed unopposed estrogenicity in the endometrium of baboons evidenced by histology, immunohistochemistry as well as circulating levels of serum estrogen. Even though the baboon model of endometriosis is a well characterized model of endometriosis, the rate of induced lesions with autologous menstrual implant was too low to be used as models to test interventional agents developed to treat endometriosis. In our model we have observed both animals treated with EC304 +progesterone developed endometriosis as early as 3months after menstrual implants. The progression of lesions were incremental with further laparoscopic analysis at 15 months. The lesions that progressed in one animal in this group was at an alarmingly high rate to see highly invasive endometriosis on 15^th^ month at necropsy that penetrated colon of the animal (Figure 3A panel a -c). In the control animal that received only vitamin E oil, there was a single approximately 0.2 mm dark spot on the ventral uterus, near the caudal aspect. The omentum was adhered to the caudal uterus, the right ovary and ventral colon but no definitive endometriosis was seen at necropsy (Figure 3A-a). Morphologically at necropsy, one baboon that was treated with P+EC304 showed an approximately 1.5×1.5×2 cm area of fibrous adhesions containing 1-2 mm orange-tan foci was present adhered to the right oviduct, uterus, and colon (Figure 3A-c). Microscopic analysis revealed endometriosis in serosa, oviduct, colon and uterus of this animal. While the second animal in the same group showed moderate adhesion of the omentum to the cranial uterus. Three masses were present in the omentum, one cranial and right of uterus, a larger mass just left of midline cranial to uterus, and one cranial and left of uterus (Figure 3A-d). The center mass extended to and adhered to the colonic wall. There were mild adhesions between dorsal mid-uterine body and ventral colon. Microscopically, there were multifocal accumulations of well differentiated endometrial glands, stroma and hemosiderin on the surface of the uterus, colon, and urinary bladder, as well as within the peritoneal cavity; large areas of hemorrhage and fibroplasia were also seen in these areas. (Using baboons as the model, this study is expensive. Hence preforming study in large groups of animals to get statistical significance is not feasible).

The animals treated with progestin alone group (n = 2) appeared essentially normal; briefly, microscopic evaluation showed multiple glands consisting of cysts lined by columnar epithelium and densely cellular stroma with mild infiltration by lymphocytes and eosinophils on the serosa, and mildly infiltrating the urinary bladder wall. The urinary bladder propria-submucosa is markedly infiltrated by eosinophils and fewer lymphocytes, with some eosinophils transmigrating the epithelium. However these animals were noted for random bleeding signs and later on spotting. The observed bleeding irregularities may be due to the effect of progesterone treatment, as noted in women who use progestin-alone contraception or are undergoing treatment for endometriosis using pure progesterone receptor agonists.

Fazleabas et al. showed that development of progesterone resistance is a gradual process and becomes evident after 6 months of disease induction [29]. Our study suggests that depleting progesterone levels by treatment with a novel antiprogestin, EC304 in the endometrium will increase estrogen hyperresponsiveness that leads to increased endometriotic lesion progression in the baboon model. Furthermore, progesterone mRNA levels were not increased in those specimens treated with antiprogestin. This may be due to the fact that established endometrial lesions show progesterone resistance.

**Figure 4:**
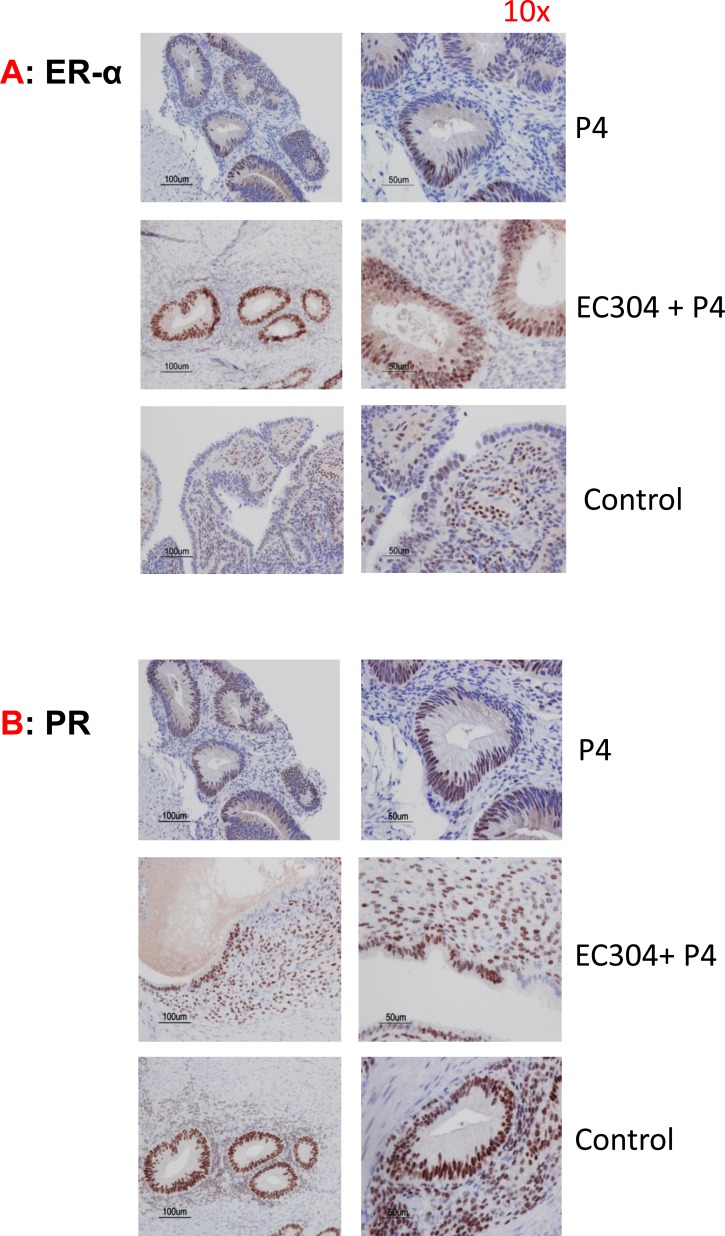
Levels of steroid receptors in antiprogestin treated baboons **A.** Estrogen receptor-alpha (ER-α) levels were elevated in endometrium of baboons treated with P+ antiprogestin (EC304) due to unopposed estrogenicity. **B.** Progesterone (PR) levels relatively lowered or unchanged in animals treated with antiprogestin.

**Figure 5:**
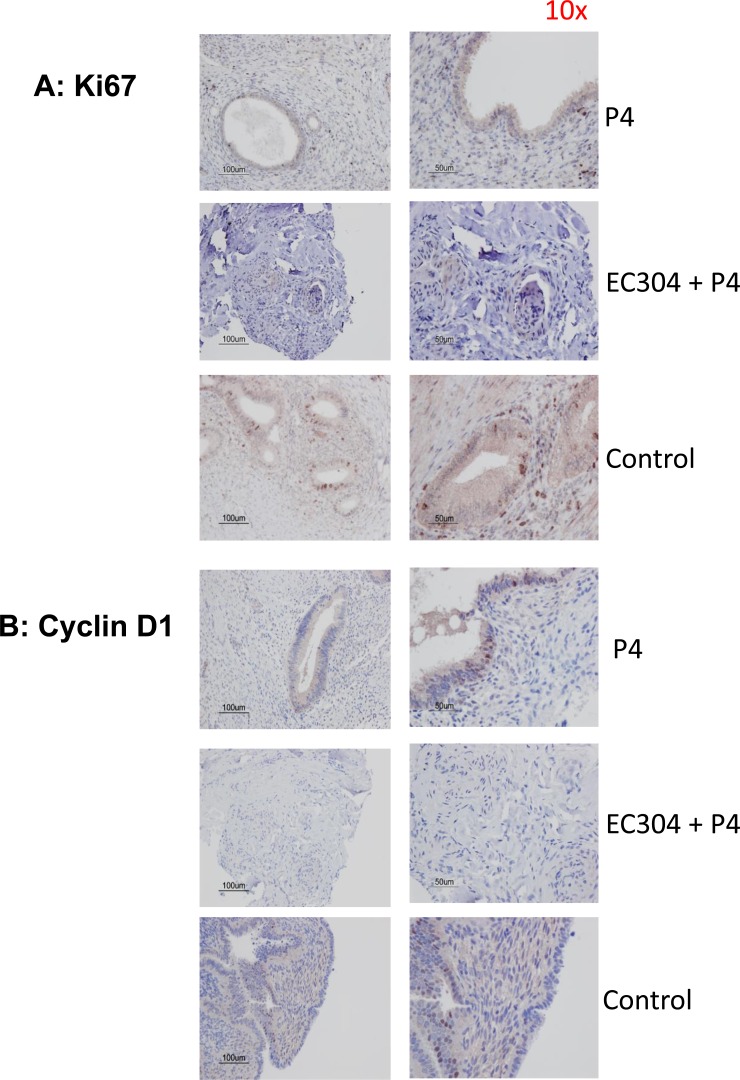
Levels of proliferation markers in antiprogestin treated baboons **A.** Ki67 levels were elevated in endometrium of baboons treated with P+ antiprogestin (EC304) due to unopposed estrogenicity driven by ER-α. **B.** Cyclin D1 levels relatively unchanged in animals treated with antiprogestin eventhough we have seen cyclin D1 has increased at mRNA level.

Immunohistochemistry analysis of the tissue collected from animals that received EC304 + progesterone clearly showed elevated expression of estrogen receptor-alpha (ERα) as direct evidence of increased estrogenicity in the endometrium that provided the initiation of cell proliferation (Figure 4). Proliferation makers such as Ki67 was up-regulated in the specimens treated with antiprogestin EC304 as result of unopposed estrogenicity (Figure 5). However, cyclin D1 levels relatively unchanged in animals treated with antiprogestin eventhough we have seen cyclin D1 increased at the mRNA level. We speculate that this increased mRNA stability did not increase the translation, or perhaps increased degradation while keeping protein levels at a steady state.

**Figure 6:**
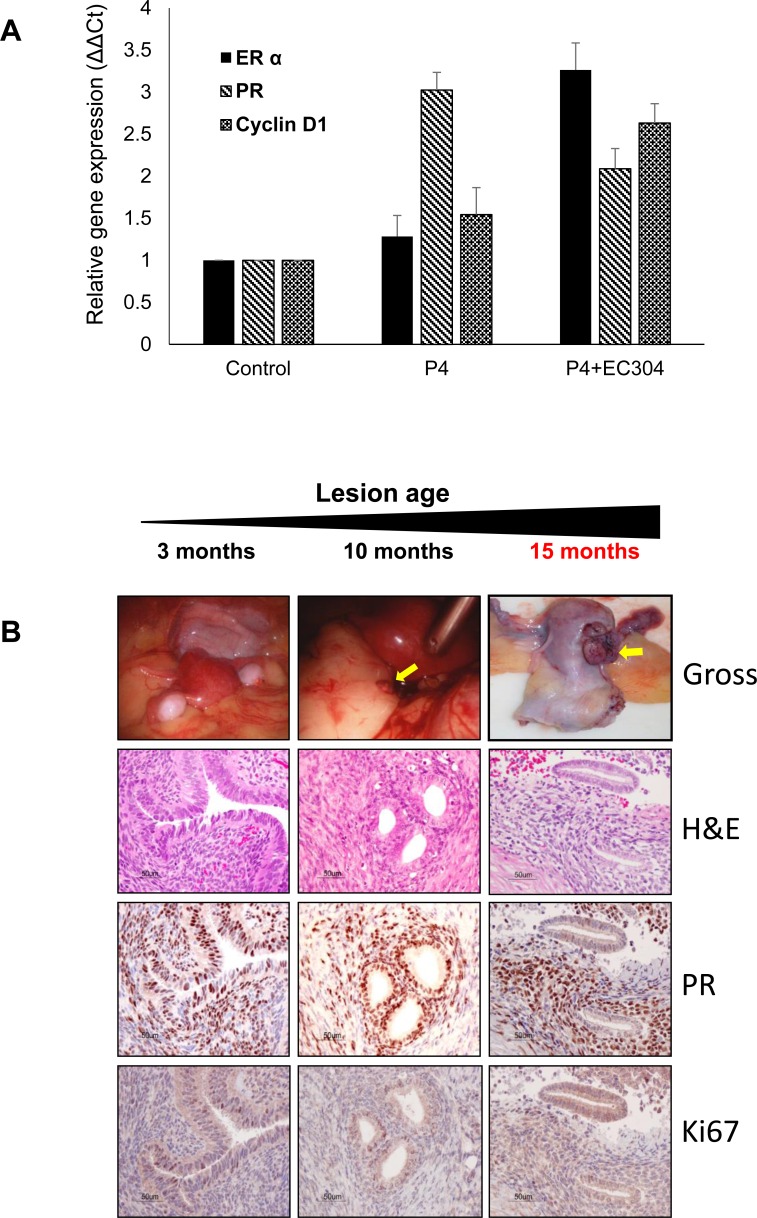
Gene expression of steroid receptors in the baboon endometrium treated with progesterone and antiprogestin (EC304) **A.** mRNA expression of estrogen receptors (ER and PR) was elevated in the endometrium of baboons treated with P+EC304 when compared to progesterone alone treated group. The increased expression of ER induced proliferation is further evidenced by increased expression of cyclin D1. **B.** Gradual progression of endometriotic lesion was analyzed by histology and immunohistochemistry. Levels of PR remained unchanged, however the proliferation marker such as Ki67 showed unopposed estrogenicity induced by antiprogestin treatment.

These data correlated with our observations from gene expression studies at mRNA level (Figure 6A). However, the PR the mRNA levels were not increased in those specimens treated with antiprogestin; this may be due to the fact that established endometrial lesions show progesterone resistance. In order to determine the level of progesterone receptors in the lesions during the progression of the disease, we performed another immunohistochemistry analysis in lesions that were collected during laparoscopies performed at 3 and 10 months and endometriotic tissue collected at necropsy. This experiment revealed steady levels of PR expression in those gradual lesions (Figure 6B).

Estrogen ELISA showed the change in the serum levels of estrogen in the antiprogestin treated animals were higher than that of control and progesterone alone groups (Figure 3B).

## DISCUSSION

We report an *in vivo* baboon model for creating early and progressive endometriotic lesions that allow testing of interventional drug candidates developed to treat endometriosis. We have also conducted *in vitro* studies to mimic early endometrial attachment to peritoneal mesothelium to assess the role of unopposed estrogenicity, however the *in vitro* model did not show a correlation with *in vivo* data due to lack of real time physiological interaction of endometrial epithelial and stromal interactions. Nevertheless, our *in vitro* and *in vivo* studies suggest that endometrial epithelial attachment to peritoneal mesothelium and subsequent trans-mesothelial invasion is a rapid process that may not be recapitulated in an *in vivo* experimental set up. On the other hand, the induction of unopposed estrogenicity is a relatively slow process to be reiterated *in vitro*. Endometrial tissue growing on the peritoneal surface has been witnessed *in vivo* in baboon models previously, however the rate of spontaneous endometriosis with autologous menstrual implants has been found to be as low as <30% [16]. The relevance of our improved model of endometriosis by induced unopposed estrogenicity will not only increase the incidence of endometriosis to study the pathophysiology of the diseases but also to test the efficacy of the drug candidates to treat endometriosis. Even though clinical evidence of the progression of endometriosis to hyperplasia and adenocarcinomas are rare, the presentation of adenocarcinoma in one of the animals treated with antiprogestin + progesterone in our study reinforces the relevance of estrogen dependence in endometrial hyperplasia and adenocarcinoma in concordance with published literature [28].

It has been widely accepted that the use of exogenous estrogens influence the development of endometriosis as well as endometrial cancer. This effect appears to be less if estrogens use along with progestagens [30, 31, 32]. However, progestin-only contraceptives such as medroxyprogesterone acetate (MPA) or PR agonists such as Mirena™ often cause bleeding irregularities and vaginal dryness due to complete suppression of estrogenic microenvironment in the uterus and endometrium [33, 34]. On contrary the use of both natural and conjugated estrogen for hormone replacement therapy (HRT) increases the chances of endometriosis as well as occult tumors in breast tissue that might increase the predisposition to breast cancer in later life [35, 36]. Hence an attractive alternative would be an ideal selective progesterone receptor modulator (SPRM) or so-called mesoprogestin that would control the bleeding irregularities of Mirena™ or similar drugs without inducing unopposed estrogenicity in the endometrium would be beneficial to treat endometriosis and perhaps early stage endometrial cancer. A recent review of epidemiological and molecular data strongly suggest that endometriosis might be the point of genesis of ovarian clear cell adenocarcinoma (OCCA) and endometrioid adenocarcinoma (OEA) [37]. However, as they do not fully address the nature of the presumed progression in humans, our model would be a better tool to study the disease progression stages.

As suggested previously in the results, mRNA levels were not increased in the study specimens treated with antiprogestin may be due progesterone resistance. Progesterone resistance induces side effects such as bleeding irregularities and vaginal dryness and also endometrial cancer [23]. It has been shown that SPRMs can very rapidly interrupt heavy uterine bleeding and allow reduction of fibroid uterine volume by 40-50% [24]. Hence apart from endometriosis an attractive area to utilize our model would be uterine fibroids.

In summary, the present study describes a baboon model of endometriosis induced by unopposed estrogenicity. Even though the number of animals used was lower than necessary to reach a statistically significant conclusion, this study might open up a possibility for improving the existing baboon model of endometriosis to conduct proof of concept efficacy studies for the treatment of this enigmatic disease. Also observations from the present study enable us to modulate the induced unopposed estrogenicity and treat using our rationally designed mesoprogestin candidates. This approach may result in the fine tuning of endometrium to get a better understanding of the pathogenesis of endometriosis and its treatment.

## MATERIALS AND METHODS

### Cell lines

The *in vitro* experiments were conducted at Evestra, Inc. Human endometrial stromal cells (HESC) (ATCC, Manassas, VA) and peritoneal mesothelial cells (LP9) (kind gift from Dr. Robert Schenken, University of Texas Health Science Center at San Antonio) were cultured in RPMI medium containing 10% fetal bovine serum (FBS) (Invitrogen, Carlsbad, CA, USA) supplemented with glutamine.

### Animals

All baboons (*Papio anubis*) were obtained from the Southwest National Primate Research Center at the Texas Biomedical Research Institute (Texas Biomed) in San Antonio, TX, USA. All procedures and care were approved by the Institutional Animal Care and Use Committee.

#### a Role of progesterone in the attachment of endometrial stromal cells to peritoneal mesothelial cells

To evaluate the role of progesterone in facilitating the attachment of human endometrial stromal cells (HESCs) to peritoneal mesothelial cells (LP9), we determined the attachment of HESC on the monolayers of LP9 in the presence and absence of EC304 or progesterone. Differences in the rate of invasion of endometrial cells through the coated membranes with and without an overlying monolayer of LP9 could be dependent on differences in the rate of attachment of HESC to LP9 cells as described elsewhere [3].

In brief, the confluent HESCs were harvested, washed with PBS and labeled with Cell tracker green (Molecular Probes; 5 mM) for 20 minutes at 37°C. The HESCs were plated at 20,000 cells per well over 96-well plates with confluent LP9 grown over growth factor reduced-matrigel (GFR-MTGL) or GFR-MTGL alone. Plates were treated with progesterone (0.001, 0.01, 1 and 10nM) and or antiprogestin (EC304). Plates were then cultured at 37°C for 1 hour in 5% CO_2_ in air. The plates were inverted, submerged in a bath of phosphate-buffered saline containing calcium and magnesium (Life technologies, Grand Island, NY), and incubated at 37°C in 5% CO_2_ in air for 15 minutes on an orbital mixer (Barnstead/Thermolyne, Dubuque, IA) set at 20 rpm, allowing non-adherent endometrial cells to segregate under gravity. Fluorescence readings were taken for each well before and after washing. Each assay was run a minimum of five replicates. Each data point was calculated as an average of the five replicates. The percentage of attached endometrial cells was calculated for each well ([fluorescence value after washing/fluorescence value before washing] - 100). Rates of HESC attachment to LP9 cells was compared as described elsewhere [3].

#### b Effect of EC304 on the invasion of menstrual endometrial stromal cells into endometrial epithelium *in vitro*

To evaluate the role of EC304 in the initiation of endometriosis *in vitro*, we used the trans-epithelial modified invasion culture model as described earlier [3, 4]. Briefly, endometrial stromal (HESC) cells (equivalent to menstrual endometrial stromal cells) were seeded onto a monolayer of peritoneal mesothelial (LP9) cells in growth factor reduced invasion chambers (BD Biosciences) as a model of menstrual endometrial stromal cell attachment to peritoneal mesothelium and trans-mesothelial invasion. HESCs were labeled with 10mM cell tracker green (Molecular Probes, Eugene, OR) for 10 minutes and washed in PBS two times before seeding. Labeled HESCs were placed over the confluent LP9 monolayers (25,000 cells per invasion chamber). Cultures were interrupted at 1, 3, 6, 12, and 24 hours by placing the invasion chamber in cold 4% paraformaldehyde. Membranes were detached from the invasion chambers by cutting them out carefully with a sharp scalpel, placed between coverslips, and examined with fluorescent microscopy. The number of invaded cells on the bottom of the coated membranes were considered as invaded through the monolayers of LP9, determined with the fluorescence microscope. Images were obtained from 8 standardized, non-overlapping fields representing approximately 40% of the total surface area.

### Modified baboon endometriosis model by induction of unopposed estrogenicity

In order to determine the effect of antiprogestin EC304 was used to induce the unopposed estrogenicity to enhance the incidence of experimental endometriosis, we used the established baboon model of endometriosis [21]. Briefly, adult (6-10 year old) animals (n = 5) were treated with progestin (progesterone) alone (n = 2) or treated with progesterone+ antiprogestin EC304 (n = 2); one animal served as control. Menstrual endometrium was harvested on day 2 of two consecutive menses using a pipelle (Unimar Inc., Neuilly-En-Thelle, France) [21].

The collected menstrual endometrium in the pipelle was kept on a sterile cloth placed on a temperature regulated heated pad until deposited under laparoscopic guidance into the Pouch of Douglas, the broad ligaments adjacent to oviducts and the ovaries using the pipelle. Laparoscopy was used to visually confirm the absence of any lesions before the collection of menstrual endometrium. Baboons received progesterone (30mg/day for 5 days a week for 3 weeks) orally after depositing the menstrual endometrium. The progesterone dose was chosen based on the fact that an average woman produces about 30 milligrams daily during the first two weeks of pregnancy. Antiprogestin (EC-304 (20mg)) was administered 5 days per week from day 1 of menstrual endometrial implantation until day 15. The control baboon received only vehicle (vitamin E oil, Naturemaid). Subsequent laparoscopies were performed at 3, 6 and 10 months after implantation during the window of uterine receptivity (day 9-11 post-ovulation in baboon). An intravenous (i.v.) catheter was surgically placed in all animals for blood collection. During laparoscopies, the number, color and position of each visible lesion was documented by video recording. Biopsies of all lesions were collected during laparoscopies. Animals were sacrificed at the 15th month after menstrual endometrial implantation and all lesions were dissected from surrounding tissue. One half of each biopsy or necropsy sample was collected for histopathology. The other half was snap frozen and stored until use at −80°C. RNA was isolated from these tissues and ER and PR target gene expression was analyzed using real time PCR.

### Histology and immunohistochemistry of endometrial samples

Histopathology samples were fixed in 10% neutral buffered formalin, processed conventionally, embedded in paraffin, sectioned, stained with hematoxylin and eosin and evaluated by board certified veterinary pathologists (M.O, E.D), who were blinded to treatment groups.

**Scheme 1:**
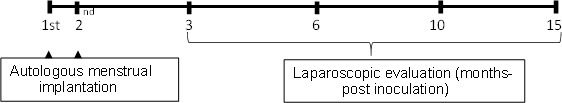
Experimental model of endometriosis in baboons, design and timeline

Routine (Scheme. 1 immunohistochemistry was performed for estrogen receptor, progesterone receptor, Ki67 and cyclin D1. Paraffin embedded sections were cut using a Microm rotary microtome at a thickness of 3 microns and mounted on positively charged slides. Sections were deparaffinized in xylene, rehydrated in graded alcohols, and rinsed in distilled water prior to antigen retrieval. Sections were then immersed in Dako Target retrieval solution, pH9, in a plastic coplin jar and heat induced epitope retrieval was achieved using a decloaking chamber (Biocare Medical). Upon removal from the decloaking chamber, approximately half of the target retrieval solution was replaced with room temperature distilled water and sections were allowed to cool for a minimum of 10 minutes before proceeding. Following the cool down period, sections were thoroughly rinsed in distilled water followed by Dako wash buffer. Sections were then treated with Dako Dual Endogenous Enzyme Block for 10 minutes at room temperature in a dark, humid chamber. (The method of incubation remains constant for the remaining steps of the procedure.) Following the enzyme block, sections were rinsed with buffer and incubated with primary antibody for 30 minutes. The antibodies tested include PR (H-190) (Santa Cruz Biotechnology, sc-7208), PR-B(C1A2) (Cell Signaling Technology, #3157), ERα(H226) (Santa Cruz Biotechnology, sc-53493), Ki-67(H-300) (Santa Cruz Biotechnology, sc-15402), and Cyclin D1(92G2) (Cell Signaling Technology, #2978). Detection of the primary antibody was achieved using Dako Advance HRP system. After rinsing with buffer, sections were covered with Advance HRP Link and incubated for 30 minutes. Sections were then rinsed again with buffer and allowed to incubate with Advance HRP Enzyme for an additional 30 minutes. While the sections incubated, the Dako Liquid DAB+ Substrate Chromagen System was prepared by adding one drop of the DAB chromagen to 2 mls of the substrate buffer. Following another wash buffer rinse, the sections were covered with the DAB solution and incubated for approximately 2-4 minutes to form a brown end product at the site of the target antigen. Sections were quickly rinsed in distilled water to halt the reaction. Counterstaining was achieved using Statlab's Reserve Hematoxylin solution. Sections were then dehydrated, cleared and mounted for microscopic evaluation.

### Reverse-transcription PCR

Real-time quantitative RT-PCR RNA was extracted from cells using a TRIzol (Sigma)/chloroform method and cDNA was synthesized using the ImProm-II Reverse Transcription System (Promega) and an oligo(dT)15 primer. Quantitative real time reverse transcription PCR (RT-PCR) was conducted in triplicate using an ABI prism real-time PCR (MJ Research) alongside no-template controls. Each 25 μL reaction included 12.5 μL DyNamo master mix (Finnzymes) and 10 pmol of primers. The relative levels of p21, p27, cyclin D1, and glyceraldehyde-3-phosphate dehydrogenase (GAPDH) transcripts were calculated using the ΔCT method [26]. The primers used were the following:

GAPDH: GGCCTCCAAGGAGTAAGACC (forward)
AGGGGTCTACATGGAAACTG (reverse)
Cyclin D1: ACGAAGGTCTGCGCGTGTT (forward)
CCGCTGGCCATGAACTACCT (reverse)
ER: TGTGCAATGACTATGCTTCA (forward)
GCTCTTCCTCCTGTTTTTTA (reverse)
PR: ACAGAATTCATGAGCCGGTCCGGGTGCAAG (forward)
ACAAGATCTCCACCCAGAGCCCGAGGTTT (reverse)

### Determination of estrogen level in the circulation of EC304 treated baboons

The amount of estradiol (E2) in the serum was measured by ELISA kit (Calbiotech, Spring valley, CA) based on the principle of competitive binding between E2 in the test specimen and E2-enzyme conjugate for a constant amount of anti-estradiol conjugated polyclonal antibody [27]. Anti-E2 coated wells were incubated with E2 standards, controls, samples and E2 enzyme conjugates. A fixed amount of HRP- labelled E2 competes with the endogenous E2 in the standard or samples. E2 peroxidase conjugate immunologically bound to the well progressively decreases as the concentration of E2 in the specimen increases and that will be measured spectrophotometrically at 450nm. The mean absorbance value for each specimen was used to determine the corresponding concentration of E2 in pg/ml from the standard curve.

### Statistical analysis

The number of invaded HESCs through MTGL- and GFR-MTGL–coated membranes was compared in the presence and absence of a confluent monolayer of LP9s, using Student's *t*-test. As well, the percentage of attached ESCs to GFR-MTGL and confluent LP9 grown on GFR-MTGL was compared by using Student's *t*-test. The *in vitro* experiments were repeated independently three times in quadruplicates and values were represented as standard deviation (SD).
